# ExpoLib: a framework for an MS/MS exposome library of anthropogenic and natural toxicants and their biotransformation products

**DOI:** 10.1007/s11306-026-02481-x

**Published:** 2026-08-26

**Authors:** Vinicius Verri Hernandes, Miguel A. Aguilar Ramos, Rolf Breinbauer, Philipp Fruhmann, Hannes Mikula, Monika Ehling-Schulz, Ellen L. Zechner, Emily P. Balskus, Benedikt Warth

**Affiliations:** 1 Department of Food Chemistry and Toxicology, Faculty of Chemistry, University of Vienna, Währinger Straße 38, 1090 Vienna, Austria; 2 Exposome Austria, National Research Infrastructure and EIRENE-AT Node, Vienna, Austria; 3 Department of Chemistry and Chemical Biology, Harvard University, 12 Oxford Street, Cambridge, MA 02138 USA; 4 Institute of Organic Chemistry, Graz University of Technology, Stremayrgasse 9, 8010 Graz, Austria; 5 Institute of Applied Synthetic Chemistry, TU Wien, 1060 Vienna, Austria; 6 Department of Physical Chemistry, Faculty for Chemistry, University of Vienna, Wahringer Straße 42, 1090 Vienna, Austria; 7 Institute of Microbiology, Centre of Pathobiology, Department of Biological Science and Pathobiology, Vetmeduni, Vienna, Austria; 8 Institute of Molecular Biosciences, University of Graz, Humboldtstrasse 50/I, 8010 Graz, Austria

**Keywords:** Exposure and effect analysis, High-resolution mass spectrometry (HRMS), Endocrine disrupting chemicals, Suspect and non-targeted screening, Structural elucidation, Microbiome-related genotoxins, (Xeno-)metabolomics

## Abstract

**Introduction:**

Despite technological advancements over the last three decades in small-molecule omics, compound annotation remains a major bottleneck in untargeted metabolomics and non-targeted environmental analysis. This is especially true for exposomics applications, which remain significantly affected by the limited chemical space coverage.

**Objectives:**

This work aims at describing the development of an MS/MS spectral library containing > 170 relevant xenobiotics from different classes of food, environmental, and microbial toxicants.

**Methods:**

LC–MS/MS data was acquired using collision-induced dissociation in data dependent acquisition mode under 13 different single collision energies with four additional collision energy spread experiments. A diverse set of compounds including natural toxins produced by bacteria, fungi (mycotoxins), and plants (phytotoxins), as well as anthropogenic chemicals such as bisphenols, phthalates, PFAS chemicals, drugs, consumer care products ingredients, and pesticides, and additional toxicologically relevant chemical classes were screened. Metabolic products for which commercially available reference standards and/or MS/MS spectra are not available in any public or commercial database have been included (e.g. colibactin-DNA-adduct, cereulide, deoxynivalenol-3-glucuronide). Library generation was performed in mzmine.

**Results:**

Open-format data based on representative spectra are provided.This new resource, available at https://zenodo.org/records/20715576, is aimed at providing a ready-to-use tool for the annotation of key exogenous compounds which are frequently overlooked in clinical metabolomics but may exert potent biological effects. A detailed discussion from a user perspective is provided regarding the library generation workflow in mzmine, aiming at facilitating the work of fellow researchers in the creation of their own in-house libraries.

**Conclusion:**

We intend to provide the metabolomics community with better tools for exposomics research and to reduce perceived barriers in developing specialized MS/MS libraries for widening chemical space coverage and increasing quality and confidence.

**Graphical Abstract:**

**Figure:**
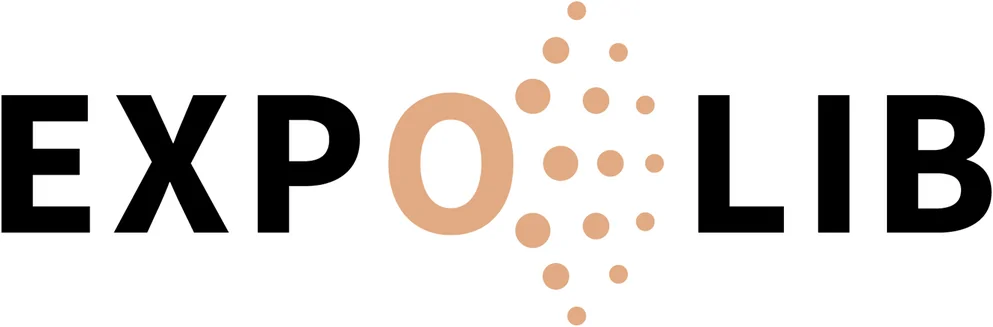

**Supplementary Information:**

The online version contains supplementary material available at https://doi.org/10.1007/s11306-026-02481-x.

## Introduction

In spite of the recent developments and the rising importance of in silico tools (Egede Frøkjær & Hansen, 2025; Hoffmann et al., 2022; Schymanski et al., 2021) for compound annotation in small-molecule omics, the use of experimental MS/MS spectra from reference standards is still considered the gold standard for compound annotation, providing with the highest annotation confidence. Consequently, in the absence of an *in-house* reference standard for unambiguous compound identification by retention time and accurate mass matching, researchers typically rely on the use of open-source mass spectral fragmentation libraries for annotation.

The field of metabolomics has recently benefited from a number of initiatives, including the Human Metabolome Database (Wishart et al., 2007, 2022), the METLIN database (Warth et al., 2017; Xue et al., 2020), the Global Natural Product Social Molecular Networking (Wang et al., 2016) (GNPS), or MassBank (Horai et al., 2010; Neumann et al., 2025), which all contributed to increase MS/MS spectra availability via spectral databases. Similarly, recent collaborative work across the MassBank of Europe, the NORMAN network, the US Environmental Protection Agency (EPA) and the US National Institute of Health (NIH) have significantly contributed to the development of open-source MS/MS libraries focusing on specific areas in exposomics (Elapavalore et al., 2023; Schulze et al., 2012; Ulrich et al., 2019).

There is a great need for further expanding the representativeness of the known chemical space. In PubChem, for instance, > 100 million compounds were listed by 2020, with < 75 000 (0.08%) containing available MS/MS spectra (Oberacher et al., 2020). Additionally, as collision induced dissociation, which is typically used in LC-HRMS/MS applications, tends to be less reproducible across MS platforms when compared to electron ionization-based fragmentation (typically used in GC–MS applications), enriching open-source databases with a diversity of MS platforms/vendors and or experimental conditions can potentially enhance annotation performance. Moreover, many small molecules are subjected to ionization in both positive and negative polarity. As the majority of NTA studies based on LC-HRMS are typically performed using both polarities to increase chemical space coverage, it is therefore expected for many compounds of interest to be detected in both datasets. Clearly, more comprehensive libraries covering both polarities are needed, as many of the compounds with currently available MS/MS spectra tend to include only the most efficient ionization mode. Finally, the lack of data curation and the underreported metadata from available libraries (such as RT) strongly limit their use for broader applications, such as the development of RT time prediction models.

This works provides the first version of the ExpoLib MS/MS library covering a representative set of highly diverse and partially commercially non-available exogenous compounds and additional metabolites. The selection of compounds was based on toxicological relevance, which is also reflected by their inclusion in human biomonitoring programs and policies. We aim at supplying especially the metabolomics community with an extended look into key exogenous compounds, typically less represented in commonly used spectral databases. The library includes main representatives for mycotoxins, pesticides, perfluoroalkyl substances, veterinary drugs, personal care product ingredients, smoking markers, industrial side products and flame retardants, among others. It also includes previously unreported compounds when considering the main open source resources available for exposomics/metabolomics but its main contributions are the addition of spectra not available in major databases (even if available commercially); the rigorous quality control around data acquisition and processing, ensuring high quality full open-source fragmentation spectra; the inclusion of less favored ionization mode, i.e., mostly ESI− lacking in current databases; the extensive metadata (and available raw data) around the spectral library, including RT from LC-based separation; and finally, the broad range of CE employed, making this dataset valuable for in-silico prediction models. Additionally, we aim at contributing to similar developments in the field by providing a detailed user perspective on the development process, performed in mzmine. As one of the most used tools in metabolomics for data (pre)-processing, it has an equal potential to serve as a widespread tool for the development of reproducible mass spectral libraries—which is still incipient in the community so far. We describe the route taken from data acquisition to QA/QC strategies, library generation and benchmarking to ensure reliability and reproducibility under previously recommended guidelines (Oberacher et al., 2020). We include hints and pitfalls found along the process aimed at complementing the available documentation provided by the software developers by offering the perspective of less experienced users. We hope to encourage other researchers to create similar open-source spectral databases as well as to gradually increment this available resource in upcoming versions, therefore supporting a more comprehensive and accurate compound annotation in the future.

## Material and methods

### Chemicals

A single working solution of reference standards previously available in-house was used in this work. In summary, the mixture included 223 compounds from a highly diverse set of chemical properties and applications, including bisphenols, phthalates, myco- and phytotoxins, pesticides, PFAS, antibiotics, industrial pollutants and more. The complete set of compounds alongside the concentrations and additional information is provided in Table S1 (compounds #1 to #223) and has also been employed in previous work (Gu et al., 2025a, b; Hernandes et al., 2025). In addition, six compounds not commercially available or only available at limited purity were in-house synthesized or isolated. These include mostly bacterial toxins such as cereulide (Bauer et al., 2010), the colibactin prodrug and the colibactin DNA adduct (Wilson et al., 2019) as well as tilimycin and tilivalline (Dornisch et al., 2017; Glabonjat et al., 2021), along with deoxynivalenol-3-glucuronide (Fruhmann et al., 2012) (Table S1, compounds #224 to #229). Acetonitrile (LC-MS Chromasolv > 99.9%, PN: 34967), methanol (LC-MS Chromasolv > 99.9%, PN: 34966) and isopropanol (Chromasolv > 99.9%, PN: 34965) were acquired from Honeywell. Water (HiPerSolv Chromanorm HPLC-LC-MS grade, PN: 83645) was purchased from VWR Chemicals. Ammonium fluoride (eluent additive for LC-MS, > 98%, PN: 52481) was purchased from Honeywell. The ESI X500 calibration solutions were purchased from SCIEX for positive (PN: 5049910) and negative (PN: 5042913) modes of analysis.

### Data acquisition

The working solution of reference standards was analysed using an Infinity II 1290 LC system from Agilent coupled with a SCIEX ZenoTOF 7600 system equipped with a Turbo V ion source. Chromatographic separation was achieved based on an in-house method for the separation of > 200 compounds, previously optimized and validated (Gu et al., 2025b). In brief, the method relies on reversed-phase chromatographic separation employing an HSS T3 column (Waters) at 0.4 mL/min flow rate, with 5 µL of the working solution injected into the system. A detailed description of all LC-MS parameters, including data acquisition strategy is provided in Table S2. Data for the six additional compounds previously mentioned were acquired at a later stage employing the same LC-MS conditions. In this case, single compound solutions were prepared (except for a mix of tilimycin and tilivalline). For cereulide, specifically, data was acquired using FIA-MS since the employed chromatographic separation was not suitable for this compound. The FIA method is also described in Table S2, while the MS employed was the same (ESI+) except for the m/z range, which was increased from 1000 to 1300.

As the inaugural version of the ExpoLib (which is intended to undergo future updates), a broad range of collision energy voltages was employed with multiple small increment intervals. This approach was chosen to obtain detailed data on the performance of different collision energies, providing evidence for a sound decision on CE values to be prioritized in future compounds to be included. More specifically, data dependent acquisition was performed in CE range of 10–70 V using 5 V intervals, totalizing 13 data files per polarity. In addition, four files per polarity were also acquired for mixed CE (i.e. CES, collision energy spread), namely 30 ± 10, 30 ± 20, 40 ± 10 and 40 ± 20 V. In this data acquisition mode, the set MS/MS accumulation time is divided for the acquisition of three individual spectra at CE − interval, CE and CE + interval, and spectra are merged to ultimately provide the user with a single merged spectrum.

### Preliminary data evaluation

A peak-by-peak data curation using SCIEX OS 4.0 software was initially performed to set a baseline to the expected output of the newly generated library. Each individual compound was checked for retention time, peak quality and, initially classified as “*present*” or “*absent*”. For the compounds classified as “*present*”, three additional sub-classifications were created. “*Optimal*” compounds would include all fulfilling the following criteria: (1) peak height > 1000 cps; (2) precursor mass error < 5 ppm for all CE tested; (3) at least one fragmentation spectrum acquired for every CE tested; (4) no isomer present in the working solution; (5) if an isomer was present, clear peak separation. Next, “*sub-optimal*” was used to refer to compounds in which at least one of the following was observed: (1) peak height < 1000 cps; (2) missing fragmentation spectra for 1 or more CE tested; (3) non-gaussian peak shape (peak shoulder/tail) or (4) precursor mass error > 5 ppm for at least one data file. Finally, “*non-detected*/*unreliable*” compounds included compounds for which isobaric interferences could not be addressed, i.e., no baseline separation of isotopes as well as compounds with unpredicted behaviour (e.g., peak splitting with same MS/MS). For compounds classified as “*sub-optimal*” due to the lack of an MS/MS spectrum in at least one file, the total number of missing files was registered as a later quality check. Finally, a compound list including both ‘*optimal*’ and ‘*sub-optimal*’ analytes was used for library development. This filtered compound list included relevant metadata (e.g.: compound formula, InChIKey, SMILES, among others) and was defined as “*Database file”* as the term employed in mzmine. The database files for both polarities are publicly available on Zenodo (see Data availability).

### Library development using mzmine

Library generation was performed similarly as described by Brungs and co-authors (Brungs et al., 2025) using the ‘*Processing wizard*’ function in mzmine (Schmid et al., 2023) (version 4.9.0). The workflow ‘*UHPLC*’, ‘*QTOF*’ and *‘Library generation’* was initially selected. Raw data (.wiff) was uploaded and extraction parameters defined (‘UHPLC’, ‘QTOF’ and ‘Filters’). These parameters are defined as ‘*presets*’ in mzmine and are listed in detail in Table S3. Next, the database file was input alongside three commonly used open-source libraries in the metabolomics field, namely The MassBank of North America Mass Spectral libraries (LC–MS/MS Negative Mode with 47,064 spectra and LC–MS/MS Positive Mode with 99,269 spectra), the MS-DIAL libraries (ESI(+)-MS/MS from authentic standards with 16,232 unique compounds (324,191 spectra) and ESI(−)-MS/MS from authentic standards with 8887 unique compounds (44,669 spectra)), and the GNPS library (ALL_GNPS_NO_PROPAGATED, 203,190 compounds). The database file contained all compound metadata (e.g., InChi, InChikey, SMILES) retrieved from each compound CAS number using a previously developed Python workflow (Brungs et al., 2025), that was kindly run and provided by the code authors. The file was manually curated afterwards and is available for download along with the presets file (*.mzwizard*) at the Zenodo repository (see Data availability). Following the setup of the presets, a ‘*batch*’ was created, with several overlapping parameters and additional new ones (for instance, parameters to filter/flag the precursor purity are only available within the ‘*batch*’ section). Similarly to the presets, all the steps included and the parameters used for the batch file are described in Table S3 and are available on Zenodo as ‘*.mzbatch’* file. Positive and negative ionization modes were treated separately under the same parameters, except for expected adducts.

This process was performed iteratively in order to identify the most suitable set of parameters for the obtained dataset. For this purpose, an R script was implemented to obtain a summarized overview of the output library from the generated *.msp* file. This summary included a list of all compounds incorporated into the library (i.e., with at least one spectrum available), the total number of spectra included (per collision energy and adduct type) as well as a “*Yes/No*” column for the presence/absence of chimeric interference (as an output attribute from the mzmine workflow). This step provided a quick overview of compound coverage and library quality to the developer and additionally served to tackle the reasons behind mismatches. This included, possibly missing spectra (i.e., spectra from clearly detected compounds based on the preliminary data check using SCIEX OS) or potential interferences (i.e., compounds with higher number of spectra than the number of files). The R code is also available to the community and intended to work after a simple update to the file path in which the *.msp* library generated from mzmine is created. Finally, the*.msp* output file was used in the following steps of QA/QC assessment and library benchmarking and an additional *.json* file was created using the same workflow. A total of four libraries were created, namely “*ExpoLib1.0_ESI*+*_M*+*H-Only*”, “*ExpoLib1.0_ESI*+”, “*ExpoLib1.0_ESI*-*_M-H-Only*” and “*ExpoLib1.0_ESI*−”. The first includes [M+H]^+^ spectra only in ESI+, while the second additionally incorporates sodium and ammonium adducts as well as protonated forms after water loss. The third includes [M−H]^−^ adducts only while the last includes both [M−H]^−^ and [M+Cl]^−^ adducts for ESI−. All library files are open source.

### QA/QC assessment

Quality assurance and quality control measurements were implemented throughout the entire process, from data acquisition to library benchmarking. The proposed European guidelines for QA/QC of tandem mass spectral libraries (Oberacher et al., 2020) were followed, with tailored quality checks additionally implemented. In summary, eight QC measures are proposed for the acquisition of tandem mass spectral data, alongside sixteen QA measures for building tandem mass spectral libraries. A detailed description of the strategies used in this study to follow all QA/QC guidelines is described in Table S4 and further discussed in Sect. 3.3.

### Library benchmarking

The library benchmarking followed two independent steps. First, all spectra from the output library were matched against extensive and widely used open-source libraries in the metabolomics community, including the MassBank of North America Mass Spectral libraries (namely LC–MS/MS Negative Mode with 47,064 spectra and LC–MS/MS Positive Mode with 99,269 spectra), the MS-DIAL libraries ESI(+)-MS/MS from authentic standards with 16,232 unique compounds (324,191 spectra) and ESI(−)-MS/MS from authentic standards with 8887 unique compounds (44,669 spectra) and the GNPS library (ALL_GNPS_NO_PROPAGATED, 203,190 compounds). All libraries were downloaded for use on September 25th, 2025. The results were manually curated in order to classify each spectral matching output into one of the four categories: true positive (TP), true negative (TN), false positive (FP) and false negative (FN). Based on such categories, four performance parameters were calculated for each collision energy separately, based the total number of annotations per categories: accuracy, sensitivity (also referred as recall), specificity and precision. A detailed explanation on the classification criteria and calculation of performance parameters is provided in Table 1. Retention time information was not used during compound database annotation neither when benchmarking against external libraries nor when performing it against the currently developed in-house library.

**Table 1:** Annotation categories and description of performance parameters calculated for library benchmarking against external libraries and in-house annotation of spiked serum samples

Matching classification	Definition	
True positive (TP)	Correct annotation based, first, on compound formula and secondly on compound name. For cases in which a match was found for formula but not for compound name, confirmation of structure based on synonyms names was performed in PubChem	
True negative (TN)	No spectral match found, and no spectra found to be available in any of the employed spectral libraries after search on formula and confirmation on compound name/synonyms	
False positive (FP)	Mismatch between known compound identity and suggested library match. Very similar matches such as positional isomers (e.g. 3-hydroxyphenanthrene × 9-hydroxyphenanthrene) were also included in this category	
False negative (FN)	Compounds with no spectral match but for which at least one MS/MS spectrum was available in one of the libraries included in the matching process	

Parameter	About	Formula
Accuracy	The overall percentage of correct results, i.e., true positives and true negatives	$\frac{TP+TN}{TP+TN+FP+FN}$
Sensitivity/recall	Showcase the capability in providing a true match when considering the missed matches, i.e., compounds present in the database but not annotated	$\frac{TP}{TP+FN}$
Specificity	Indicates the ability to return an empty match when the correct compound is not present in the employed libraries	$\frac{TN}{TN+FP}$
Precision	Overall percentage of correct hits (TP) across all hits, i.e., positive matches. In this work, it represents the complementary opposite value to classical false discovery rate (FDR*). In other words, 80% precision indicates 20% FDR	$\frac{TP}{TP+FP}$

*Classical FDR is not employed in MS/MS spectral match for untargeted metabolomics due to the intrinsic challenge on classifying spectral matches into true and false positives. Instead, alternative methods have been proposed (An et al., 2024; Scheubert et al., 2017; Wang et al., 2018). In this work, classification into the four described categories is possible as reference standards were available for all compounds described

In a second step, compound annotation performance was assessed using data previously acquired in-house. An extract of human serum was spiked at the same concentration level as the reference standards in the working solution. The spiked sample was then analysed under the exact same LC–MS conditions as for the data acquired for the library development.

Annotation of compounds was performed under two independent conditions for comparison purpose. First, spectral match was performed against the three previously employed libraries. Next, the same search/matching parameters were applied, and the newly developed ExpoLib library was also included in addition to the open-source libraries. For this step, the full library versions were used for both ESI+ and ESI−. Retention times were only considered afterwards for manual data curation and evaluation of true positives, i.e., not as a limiting parameter during the annotation process.

## Results and discussion

### Preliminary data evaluation

From 229 analytes included in the tested analyte-panel, 172 were reported in at least one ionization mode. More specifically, 106, 28 and 95 compounds were initially classified as optimal, sub-optimal and non-detected/unreliable, respectively, for ESI−. Similarly, 84, 29 and 116 were found for ESI+ . Most non-detected/unreliable compounds had a completely absent peak, likely due to poor ionization under the investigated conditions while a few others were classified as such due to the presence of at least one additional isomer in the working solution, with no baseline separation. It is important to highlight that aiming for a mixture of standards with no mass overlap is therefore recommended. If flow injection analysis acquisition mode is chosen, refraining from including isomers in the same solution is mandatory to avoid chimeric spectra. In this work, isomers were present since a routinely used working solution with known concentrations was readily available.

Preliminary manual data curation was highly beneficial to spot problematic compounds/data files and is generally recommended. However, this process might become an overwhelming task with an increasing number of compounds. In this work, roughly 3400 peaks (~ 200 compounds × 17 CE) were manually confirmed and checked for the presence of at least one MS/MS spectrum. When developing extensive databases, checking at least 10–20% of all compounds (including different chemical classes and retention times) may be a practical compromise for selecting representative reference compounds to be checked during library development. During this step, in addition to defining compound quality (optimal, sub-optimal or non-detected/unreliable), three additional points should be assessed. The first is the total number of files per compound with at least one MS/MS. Even when setting up a suitable data acquisition method, missing MS/MS might exist for different reasons (fragmentation not triggered, retention time shifts, etc.). Although such number should be expected to be a small percentage of the total number of expected MS/MS, it provides key information for benchmarking. Secondly, assessing overall inter-sample retention time variability for each compound allows the user to estimate retention time tolerance values to be input into mzmine during the development stage. Thirdly, the precursor mass error should be monitored for each compound since it is a key parameter in data extraction. This helps in estimating an average error to be used as well as to spot potentially problematic compounds.

It is also worth highlighting that the intensity threshold was determined after manual data curation and visual inspection of MS/MS data acquired, and the perception that relatively noisy MS/MS data was present below such threshold. Regardless, a “*noise threshold*” of 100 was used during peak picking. As expected, such threshold values are instrument/vendor dependent and should be set around mzmine recommendations and user experience.

### Spectral library development: a user’s perspective

Despite the user-friendly interface in mzmine aimed at simplifying spectral library generation, we do recommend taking the development of a new spectral library as a gradual process to ensure highest possible data quality. As any workflow that involves high-resolution mass spectrometry data (pre-) processing, there are many parameters to be set and optimized, with many of them heavily impacting the outcome of the generated library. In addition, comparing a previously developed library to a new version obtained after changing a few parameters is typically not a straightforward endeavour, especially when a high number of compounds is present. Therefore, starting with a single and representative data file (e.g. CE 35 V) or a subset of compounds of interest is recommended since it speeds up the process and simplifies the output library, which ultimately helps in estimating the impact of a certain parameter in data extraction.

A critical point is the handling of the database file. This file contains all compound metadata to be added to the output library, and may include InChI, InChIKey, SMILES, PubChem ID, among others. Small non-conformities in this file can lead to unexpected errors during data processing and finding the reasons behind those errors might be a difficult task. Most typical examples during the library development were the use of non-supported characters in compound name such as Greek letters (e.g.: α-zearalanol) or apostrophes; non-condensed formula formats (e.g.: C20H12O3(CH3)_2_) and non-visible blank spaces in the end of the formula cell. In addition, it is important to highlight that neutral masses or *m/z* values are not needed at all, as they can be automatically calculated based on the compound formula/SMILES listed and the adducts selected on your mzmine workflow (Brungs et al., 2025). Nevertheless, in the *Annotation tab* of the *presets*, the field “*Use Precursor m/z*” allows for the option “*as listed in the database*”, in addition to the previously mentioned “*as calculated from neutral mass (or formulas/smiles)*”. We found this to be useful at a first stage of the method development especially for the cases in which the most abundant adduct is not the same for every single compound. For instance, aristolactam I presented a considerably higher signal as [M+NH_4_]^+^ than [M+H]^+^ in our settings. Therefore, during manual data curation, we have used the ammonium adduct as the typical one followed in our studies and, consequently, selected the same adduct (*m/z*) during the optimization of parameters. This simplifies the interpretation of results when a new library is created as opposed to the use of *m/z* calculation based on formula and selection of both [M+H]^+^ and [M+NH_4_]^+^ adducts, which results in a more complex output library to evaluate (since both adducts will be extracted for all compounds listed). Therefore, having only the most intense adduct per compound might be more interesting at this initial point. In fact, two lines can be added for the same compound in the *Database file*, with two different *m/z* values if the goal is to evaluate different adducts for only a few compounds. Once the parameters have been properly set, we recommend the use of automatic *m/z* calculation for better reproducibility and accuracy.

Another advantage of having a single *m/z* value per compound at this stage is the possibility to cross check the total number of spectra obtained per compound with the expected outcome based on the preliminary manual data curation (here, performed in SCIEX OS software). From our experience, this number will be mostly impacted by the noise threshold (at both MS^1^ and MS^2^ levels) as well as the “*Minimum number of signals*” parameter under “*Quality parameters*” of the “*Batch spectral library generation*” module of the batch creation. Here, the user should consider the trade-off between two outcomes. By selecting a minimum of 2–3 peaks for a certain MS/MS spectrum to be included in the library, the user avoids the inclusion of non-meaningful spectra (for instance, when a non-fragmented precursor only is present or when only a non-specific water loss is present). At the same time, this might also cause the exclusion of meaningful MS/MS spectra for compounds that tend to produce one or two significant fragments only. Some examples in our dataset included atrazine and bis(1-chloro-2-propyl) phosphate, which mostly produced a single chlorine fragment at *m/z* 35 in ESI− at all tested CE or acetamiprid, which either produced a single fragment at *m/z* 140 for lower fragmentation energies or the same single chlorine fragment at higher collision energies. For this reason, we have opted to keep this value at 1 and later evaluate the contribution of single fragment spectra to our library.

**Fig. 1:**
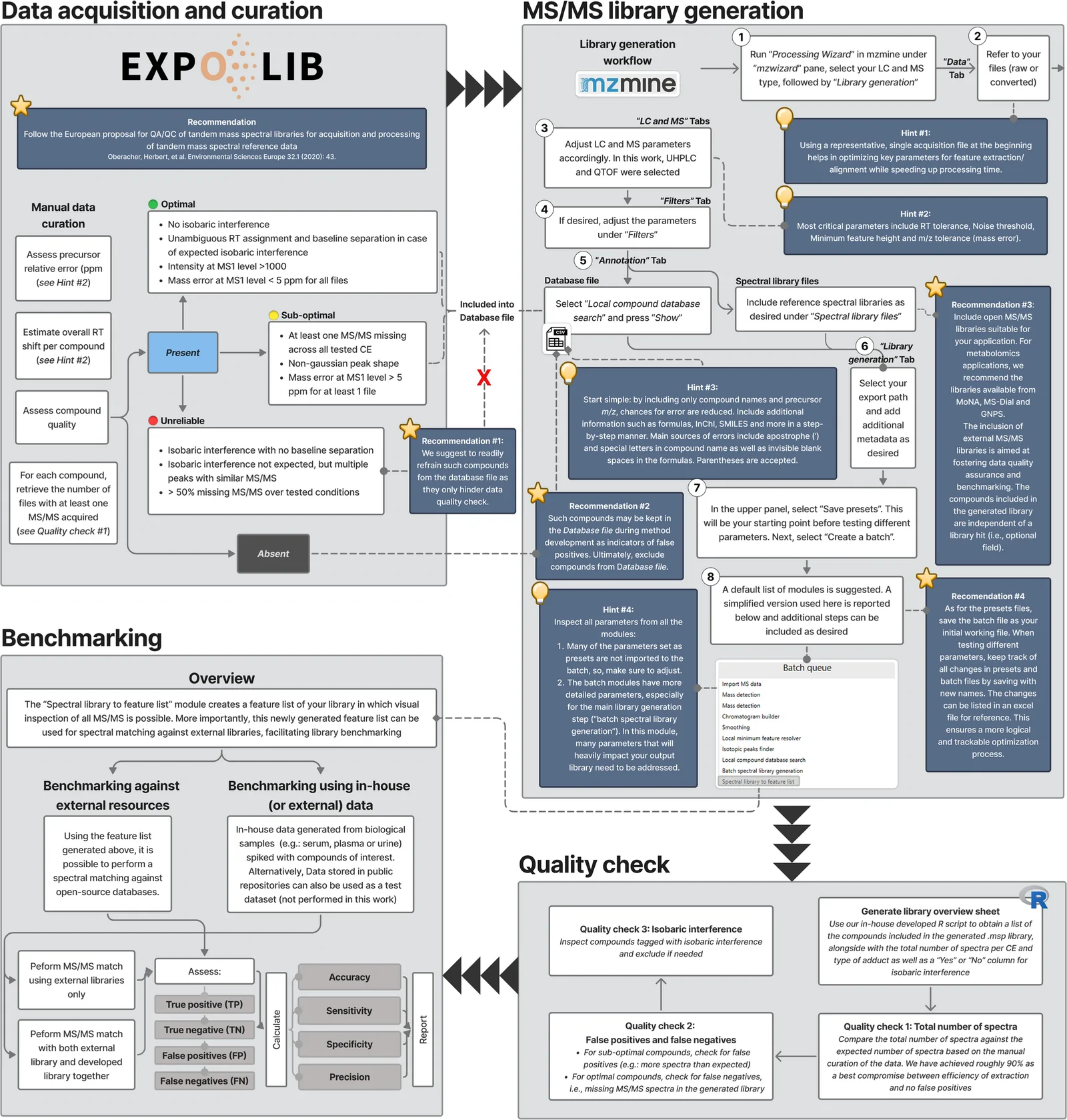
Overview of the workflow employed for the ExpoLib exposome library development, including data acquisition and curation, library generation, quality check and benchmarking. The white boxes and solid arrows represent the general workflow employed. Blue boxes depict hints and recommendations during the process. Dashed lines connect correlated steps/recommendations/hints.

A summary of the complete workflow including hints and recommendations is depicted in Fig. 1. In addition to data acquisition and curation, MS/MS library generation and quality check blocks, library benchmarking is also included and further discussed in Sect. 3.4.

### ExpoLib exposomics library overview

**Fig. 2:**
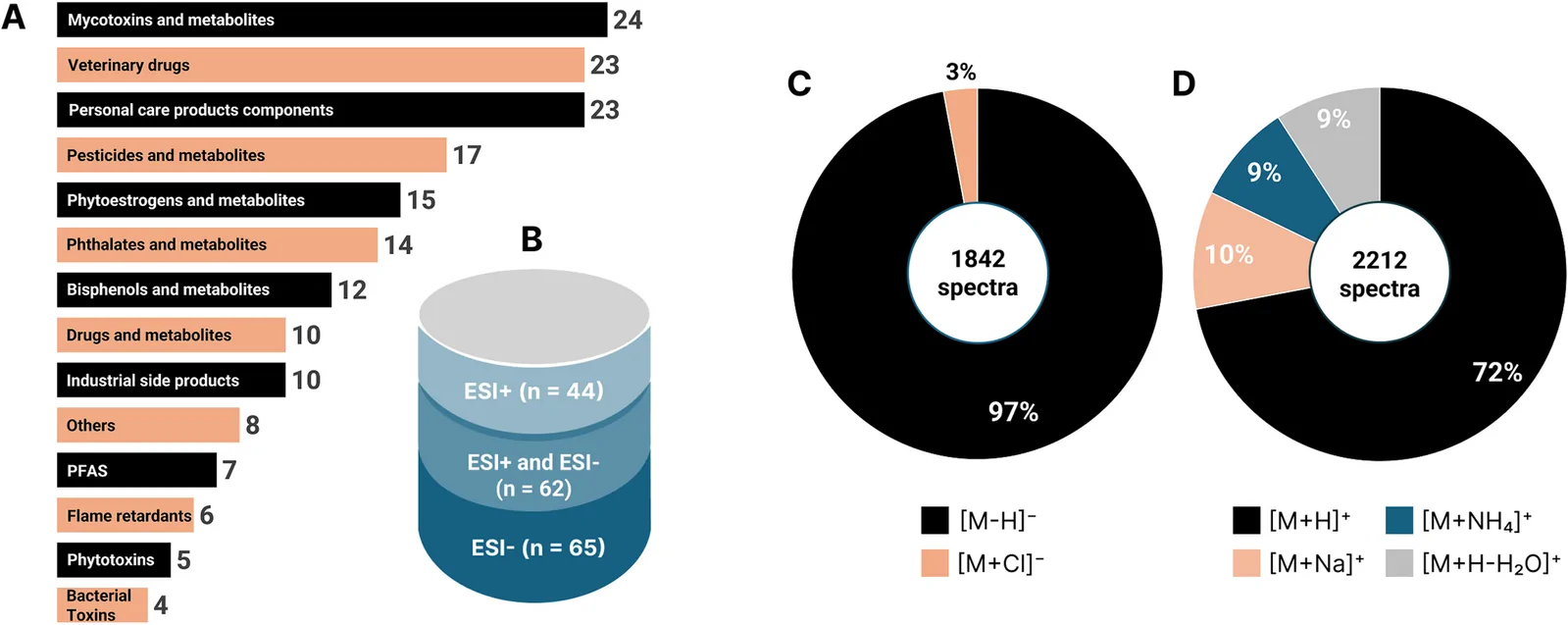
The ExpoLib in numbers: total number of compounds represented according to chemical/application classes (**A**) along with compound distribution between ionization modes (**B**). The percentage of adducts represented in the totality of MS/MS spectra is also depicted for ESI− (**C**) and ESI+ (**D**).

In total, 174 compounds were included in the developed spectral libraries. Two files were generated for negative mode, namely “*ExpoLib1.0_ESI*−*”* (containing [M−H]^−^ and [M+Cl]^−^ adducts) and “*ExpoLib1.0_ESI*−*_M-H-Only”,* containing only [M−H]^−^ adducts. Similarly, for ESI+, a full version containing [M+H]^+^, [M+Na]^+^, [M+NH_4_]^+^ and [M+H-H_2_O]^+^ adducts was created (“*ExpoLib1.0_ESI*+”) and a subset of it that includes only [M+H]^+^ adducts is also available (“*ExpoLib1.0_ESI* +*_M* + *H-Only*”). Figure 2 presents an overview of the compound classes represented (Fig. 2A), the number of compounds reported for a single polarity or both (Fig. 2B) and the distribution of adducts (Fig. 2C and D) alongside the total number of fragmentation spectra available. It is possible to observe a considerable number of compounds (> 1/3 of the total number of compounds) presenting good ionization efficiency in both ESI+ and ESI− (Fig. 2B), supporting the need for the inclusion of fragmentation spectra in both polarities whenever possible rather than focusing on the most efficient ionization mode per compound. In addition, enriching generated libraries with alternative adducts other than [M+H]^+^ and [M−H]^−^ is also of need since some compounds might present a higher ionization efficiency as adducts of sodium, ammonium and chlorine as well as being present as in-source fragments—such as water losses. Overall, it is also possible to observe the expected predominance for [M+H]^+^ and [M−H]^−^ adducts for ionization (Fig. 2C and D), with [M+Na]^+^ representing the next most frequent ion detected.

A summarized description of the full library versions (ESI+ and ESI−) is reported in Tables S5 and S6, respectively. The overview includes the list of compounds per ionization mode and a breakdown on the total number of spectra grouped by adduct type and CE.

### Spectral library benchmarking

#### Benchmarking against open-source libraries

**Fig. 3:**
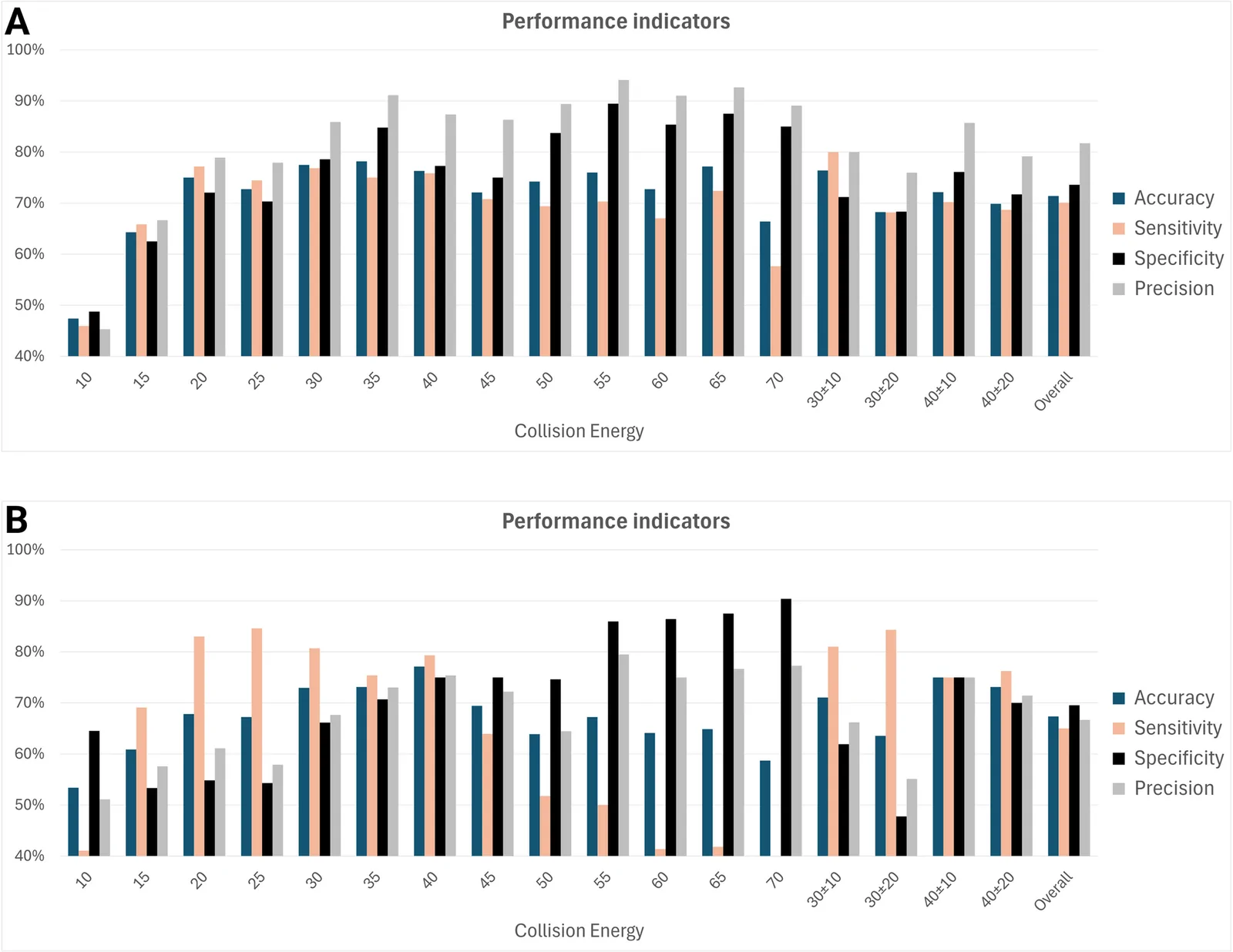
Performance indicators for the ExpoLib library in **A** ESI+ and **B** ESI− according to employed collision energy

After matching the generated spectra included in the final libraries against the selected open-source databases, the number of TP, FP, TN, FN was used to calculate performance metrics (accuracy, sensitivity, specificity and precision) for both ESI+ and ESI− libraries (Fig. 3A and B, respectively). When comparing the overall values (right-most group of bars) it is possible to observe a slightly better average performance for all metrics for positive ionization mode. In fact, this improvement in performance is especially observed for precision, which jumps from 67% in ESI− to 82% in ESI+. This indicates that the ratio of TP to FP is mainly favoured in this ionization mode, which is likely connected to the fact that ESI+ libraries tend to cover a larger chemical space, increasing the odds to obtain a TP over a FP (which might be the case especially if non-specific fragments are present).

It was observed that a slightly higher average CE in ESI− is apparently needed for achieving maximum accuracy when compared to ESI+. While in the latter CE 35 and 30 presented the highest accuracy values (78 and 77%, respectively), ESI− had increased performance at CE 40 and 40 ± 10 (77 and 75%, respectively). Nevertheless, relatively small differences in this metric are seen between CE 30 and 40 while all CES data acquired in that range showed higher performance variability, especially for ESI−, for which CE 30 ± 10 resulted in 75% accuracy while CE 30 ± 20 showed 64%. The same behaviour is observed for sensitivity, for which top ranked values include CE 30 ± 10 and 20 for ESI+ (80 and 77%, respectively) and CE 25 and 30 ± 20 for ESI− (85 and 84%, respectively). The general perception in the use of CES strategy is the acquisition of a more representative fragmentation spectra as the result of a broader collision energy span. On the other hand, there is a broad consensus within the field that such mixed spectra might result in lower matching score due to its hybrid nature and the predominance of single-CE spectra in open-source libraries. In addition, such approaches may increase total cycle time or, more typically, reduce accumulation time for individual MS/MS spectrum, leading to noisier spectra. Therefore, we aimed at evaluating the benefits and drawbacks of such an approach on compound annotation within our dataset. Based on the presented results, we can infer that the use of CES is mostly beneficial, providing similar or superior performance against the same CE value with no spread. Nevertheless, it is key to highlight that the results and metrics herein described should be carefully considered, since only a significantly small portion of the chemical space is here represented.

It was observed that in both ionization modes, CE 10 and 15 exhibited low performance, with all indicators below 70%. Therefore, despite considering this data for the descriptive analysis presented above, we have opted to exclude all spectra obtained at such CE values from the available libraries.

#### In house benchmarking: use of spiked biological samples

**Fig. 4:**
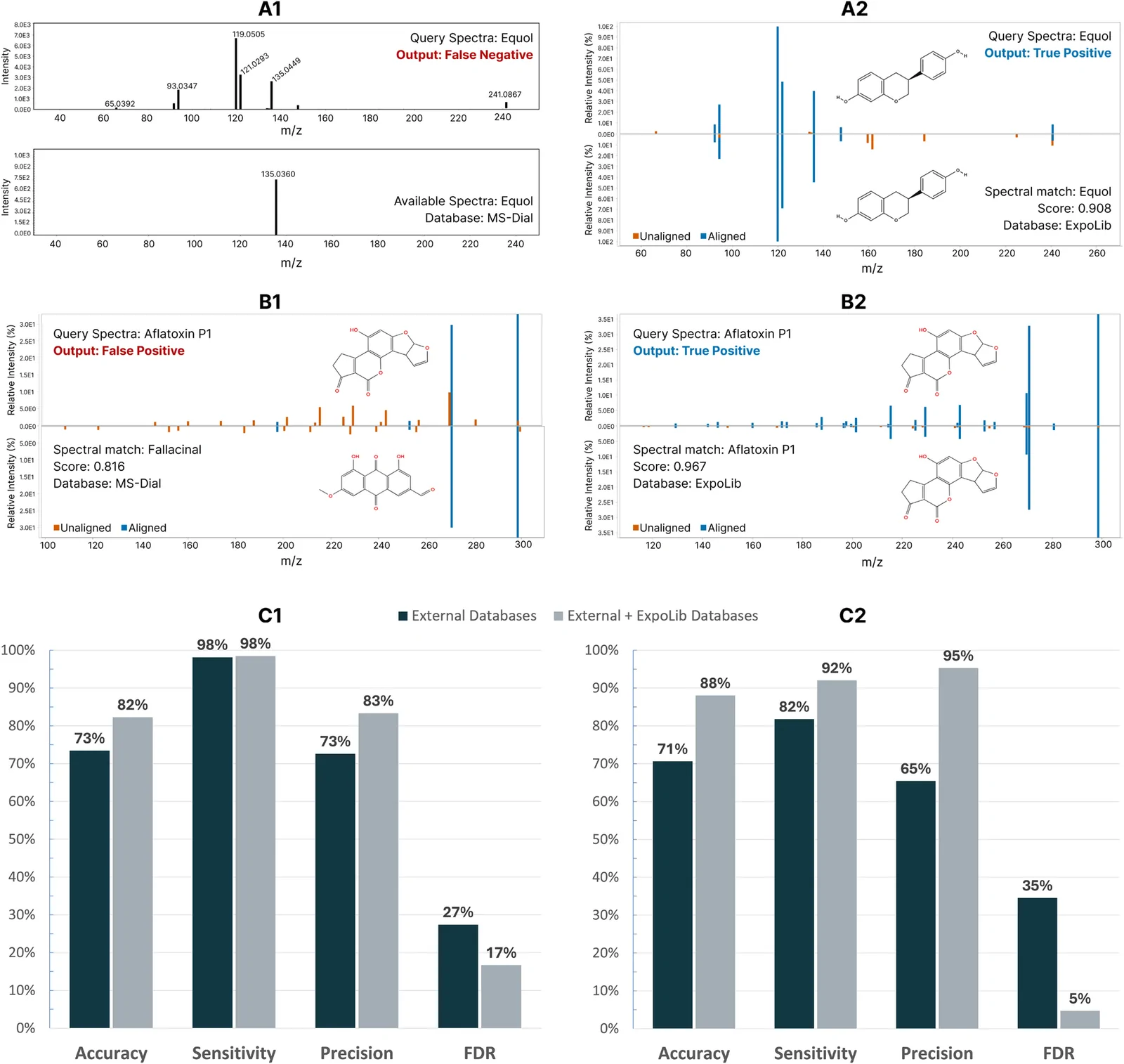
Performance indicators for library spectral matching of a spiked matrix sample (human serum) with xenobiotics covered by the ExpoLib database. A false negative annotation (**A1**) develops to a true positive (**A2**) by including the ExpoLib library. Similarly, A false positive annotation (**B1**) with satisfactory score returns a true positive (**B2**) with higher score. In summary, accuracy and precision presented a considerable increase in ESI+ (**C1**) while all performance indicators were significantly improved in ESI− (**C2**), most notably the false-discovery rate, as a consequence of the drastic reduction in false positive results

To mimic the MS/MS matching process based on open-source databases, fragmentation spectra were generated from a spiked matrix (serum) and searched against previously mentioned open-source databases. The same process was then repeated, this time with the addition of the ExpoLib to the database list. Results were compared in terms of accuracy, sensitivity, precision and FDR. FDR was chosen over specificity since this value would always retrieve zero for the second step in which the in-house library was used, once all compounds of interest are present in the library and, therefore, no true negatives is expected. Results are summarized in Fig. 4.

Overall, it was possible to observe an increased performance for all metrics in both ionization modes. As anticipated, the gaps in performance between the use of open-source databases only and the inclusion of the ExpoLib library is significantly more pronounced in ESI−, especially for precision and FDR, which are heavily dependent on the number of TP. This number substantially increases due to two factors. First and more importantly, the conversion of a large number of TN (i.e., compounds for which MS/MS spectra is not available initially) into correct matches (TP). More specifically, 29 TN were initially found, which were predominantly converted to TP. This observation reiterates the need for more comprehensive spectral databases in ESI−. Secondly, the conversion of previous mismatches (FP) to correct assignments also contributed to the overall increase in performance, with 19 initial FP hits shifted to only four.

The results obtained at this stage are described in detail in Tables S7 and S8, providing the reader with an overview of typical false annotations found for the set of compounds described in this work.

## Conclusions and recommendations

We provide a reliable resource for MS/MS-based compound annotation workflows with a special focus on exogenous compounds typically overlooked in widespread databases used by the metabolomics and exposomics communities. The resource provided covers a wide range of collision energies, chemical classes and applications as well as ionization adducts typically overlooked. As such, its application goes beyond compound annotation as a relevant resource for RT and in-silico fragmentation prediction modelling. An extensive description of compound metadata, QA/QC procedures and library benchmarking is additionally included to ensure data quality and reproducibility.

We further incorporate a detailed framework for the generation of a mass spectral library using the workflow available in mzmine, described from a user-oriented perspective to reduce the perceived complexity for first-time users. Since it is virtually impossible to find a combination of parameters during data processing and library creation that will provide the user with a library containing no missing compounds/spectra or eventually lower quality spectra, finding a good balance between compound coverage, data quality and reliable outputs should be prioritized. One of the advantages of such workflow is the reproducibility aspect, since any other user having access to the data and operational settings (“*presets*” and “*batch*” files) is able to generate the same (non-curated) library—and possibly further improve it.

Implementing and reporting QA/QC strategies should be further promoted not only during data acquisition and data processing but also for the creation and reporting performance of MS/MS spectral databases. Library benchmarking should be better explored/described in future works to illustrate the library capabilities and possible limitations. That is to say that benchmarking against in-house and/or external data should be encouraged. On the other hand, we do acknowledge the obstacles and difficulties in this process, especially related to manual data curation and performance metrics. Manual data curation of spectral matches within the context of an ever-growing number of compounds included in recently developed library is still challenging. On top of that, performance metrics—such as FDR—are still not well defined/established in the field of small molecule omics, not only due to the fact that defining a true or false positive is virtually impossible but also considering other factors such as the fact that, often times, true positive matches might not be ranked first based on matching scores.

## Supplementary Information

Below is the link to the electronic supplementary material.

Supplementary Material 1

## Data Availability

Raw data files and all ExpoLib library files are available for download in Zenodo at the Global Exposomics and Biomonitoring Lab repository (https://zenodo.org/records/20715576). Spectral libraries are available in three different file formats:*.msp* (compatible with MS-DIAL applications), *.json* (recommended by mzmine, contains further readable metadata) and .*sdf* (suitable for upload in Library View 1.8 and use in SCIEX OS software for library matching). Two versions are available for ESI+: “*ExpoLib1.0_ESI*+*_M*+*H-Only*”, limited to [M+H]^+^ adducts only, as well as “*ExpoLib1.0_ESI*+” which includes all MS/MS triggered and extracted for [M+H]^+^, [M+Na]^+^, [M+NH4]^+^ and [M+H-H2O]^+^. Similarly, for ESI−, the “*ExpoLib1.0_ESI*-*_M-H-Only*” files contain [M−H]^−^ adducts only, while the files “*ExpoLib1.0_ESI*−” compiles [M−H]^−^ and [M+Cl]^−^ adducts. For each available library, a corresponding “*Library Overview*” document follows, with a detailed description on the compounds included in each ionization mode as well as the total number of spectra per collision energy and adduct, the presence/absence of chimeric interference and the retention time. The “presets” and “batch” files used in mzmine for library generation are also available for both “*ExpoLib1.0_ESI*+*”* and* “ExpoLib1.0_ESI*−*”* libraries. Finally, we also provide the Database File employed in mzmine as a template, alongside the R-based script used to generate the “Library Overview” file.
